# How Do Stress Exposure and Stress Regulation Relate to Borderline Personality Disorder?

**DOI:** 10.3389/fpsyg.2017.02054

**Published:** 2017-11-30

**Authors:** Nadège Bourvis, Aveline Aouidad, Clémence Cabelguen, David Cohen, Jean Xavier

**Affiliations:** ^1^Service de Psychiatrie de l’Enfant et de l’Adolescent, Groupe Hospitalier Pitié-Salpêtrière, Assistance Publique Hopitaux de Paris, Paris, France; ^2^UMR 7222, Institut des Systèmes Intelligents et Robotiques, Université Paris-Sorbonne, Paris, France; ^3^Pôle de Psychiatrie Infanto-Juvénile, Centre Hospitalier Intercommunal Toulon – La Seyne, Toulon, France; ^4^Department de Psychiatrie Infanto Juvénile, Centre Hospitalier Universitaire de Nantes, Nantes, France

**Keywords:** borderline personality disorder, stress, post-traumatic stress disorder, neurovegetative activity, hypothalamo-hypophyseal system

## Abstract

Borderline personality disorder (BPD) is a severe and frequent disorder characterized by a pervasive pattern of instability affecting impulse control, emotional regulation, cognitive processing, self-image and interpersonal relationships. Patients’ personal histories are often marked by stressful or traumatic experiences, either unique or repeated. Moreover, while clinical signs of the disorder include both chronic and acute features, acute features are mostly triggered by acute stressful situations. Such features include transient cognitive distortion, intense anger, uncontrollable impulsivity, and self-harm behavior – including suicide – and contribute to the burden of the disease. In this paper, we review the various aspects (epidemiological, clinical, and physiological) contributing to the relationship between BDP and stress. In particular, we explore the statistical association between stress exposure and the emergence of BPD while taking into account other psychopathologies, such as post-traumatic stress disorder. Then, the different aspects of stress responses (namely, the phenomenological, behavioral, hormonal, neuro-vegetative and neural responses) are reviewed in BPD patients. Pathophysiological hypotheses are formulated to explain the differences in responses between BPD patients and healthy subjects and their relation to BPD symptoms. Although the pathogenesis remains uncertain, our conclusions seem to reflect a specific biological and neural pattern of altered stress perception and regulation in BPD.

## Introduction

Borderline personality disorder (BPD) is a complex personality disorder which affects 2% of the adult general population. Ten percent of psychiatric outpatients and 20% of psychiatric inpatients are diagnosed with the disorder. The associated morbidity is high, mainly due to risk-taking behavior – (such as substance abuse or reckless driving) and self-harm behavior (such as cutting or suicide attempts) (Lieb et al., 2004; American Psychiatric Association, 2013). The burden of the disorder is also related to the high mortality rate due to suicide – up to 10% of BPD subjects commit suicide, a rate almost 50 times higher than that in the general population (Lieb et al., 2004). The behavioral outbursts are often related to impulsivity, a key feature of BPD (American Psychiatric Association, 2013). Impulsivity itself in the context of BPD most often emerges in response to stressful events. BPD subjects spontaneously develop peculiar behaviors, such as self-harm, that are clearly described as stress soothing by the subjects themselves. Thus, acute stress situations are an important source of harm in BPD.

Interestingly, while personality disorders are often supposed to be structural and a stable diagnosis, cohort studies have shown that BPD is an unstable diagnosis. The historical cohort of 290 BPD described by Zanarini et al. (2016) showed that after 2 years of follow-up, 93% of subjects showed a partial symptomatic remission that led them out of the diagnosis criteria for the disease. When studied with a dimensional approach, the less stable dimensions were anger, suicidal attempts and impulsivity (Roberts et al., 2006; Blonigen et al., 2008; Zanarini et al., 2016), all behaviors that are strongly associated with stressful situations. Dialectical Behavioral Therapy, one of the most efficient therapy techniques in BPD (Linehan et al., 2015) has been shown to be more efficient on the ‘emotional regulation’ and ‘distress tolerance’ components than the ‘interpersonal relationships’ or ‘mindfulness parts’ (Neacsiu et al., 2014). Altogether, these works evoked the idea that focused therapy may be efficient in BPD (Bateman and Fonagy, 2010; Jahangard et al., 2012), and that stress-related behaviors and symptoms may be more reversible in BPD than the more chronic symptoms related to the experience of emptiness and fear of abandonment or instable relationship patterns.

Furthermore, the relationship between exposure to stress in infancy and later development of BPD in adolescence or early adulthood has been questioned for decades, starting from psychoanalytic theories which hypothesized the occurrence of a trauma during the anal stage to explain the later outburst of the borderline symptoms (Kernberg, 1967). Later, other types of early negative stimuli have been suspected to be involved, such as neglect, or invalidating environment (Linehan et al., 1994). The causal role of environmental stress in the disorder has long been debated (Paris, 1997).

To understand how stress and BDP relates, it is crucial to use an operational definition of stress. This should take into account all possibilities from dramatic trauma to chronic neglect. We therefore deliberately used a broad definition of stress that focuses on the effects or response rather than distinctions based on the nature or intensity of the stressful stimulus. We started from Hans Selye’s pioneering definition, “a non-specific response of the body to a noxious stimuli,” which we extended (or in which we included) the response of the *individual* (behavioral perspective) and the response of the *mind* (phenomenological perspective) (Rom and Reznick, 2016).

Finally, addressing the complex relations between stress and BPD is the opportunity to both tackle the issue of the etiology of the disorder, e.g., how does an early stressful environment leave its trace on the later psychological and physiological patterns of the individuals, and also the issue of the treatment, e.g., how a better understanding of the process underlying the harmful reactions to acute stress may contribute to reduce the burden of the disease.

In this work, we first focus on the literature addressing past stress exposure and BPD, crossing different approaches (psychodynamic, epidemiologic, clinical, genetic). Second, we consider the various levels of response to an acute stress in individuals with BPD, namely, the phenomenological, behavioral, hormonal, neuro-vegetative and neural levels. We finally propose a synthetic model of the response, introducing the possibility of two main profiles of acute stress response in BPD.

## Materials and Methods

**FIGURE 1:**
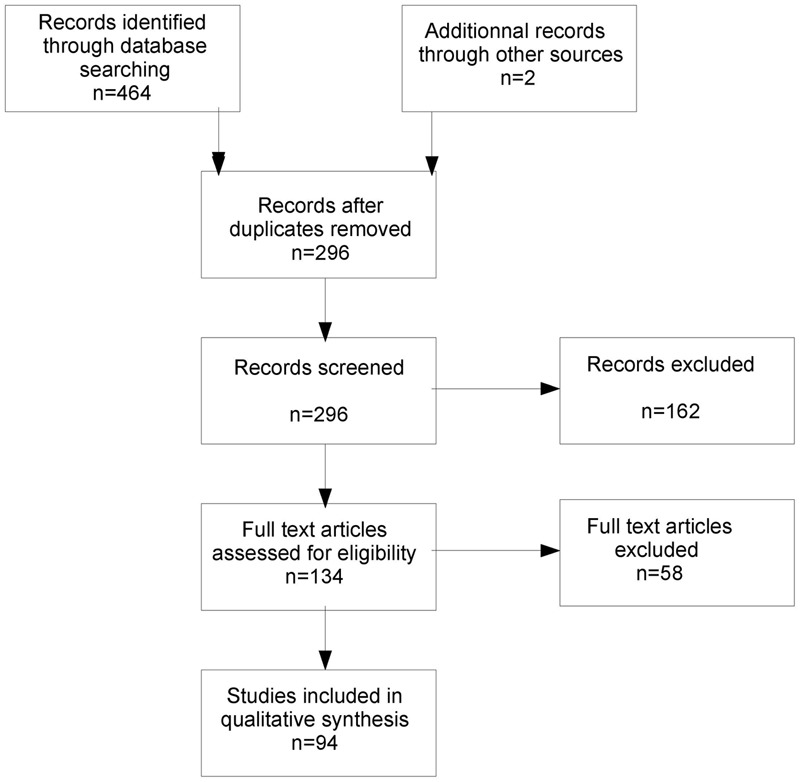
Diagram flow of the literature search.

A systematic search of relevant papers was conducted in both the Pubmed and Science Direct databases, from their start date until December 31, 2016. The database-specific keywords “stress” and “BPD” were associated and searched in titles and abstracts. For the neuroimaging review, the keywords “neuroimaging” or “MRI” were added and associated with “stress” or “negative emotion.” **Figure 1** summarizes the diagram flow. Four hundred sixty-four papers (256 in Pubmed + 212 in Science Direct) were initially found, leading to 296 papers when duplicates were excluded. One hundred sixty-two reports were immediately excluded by the two first authors of the study on the basis of the abstract content because they were not relevant to the topic of this review. One hundred thirty-four papers were fully read and qualitatively analyzed for this review. We selected the most comprehensive articles when several had been published by the same team on the same subject and mainly selected recent papers in their field when available. Finally, for the purposes of clarity and synthesis, 94 references were kept.

## Personal History of Stress Exposure and BPD

### Stress Exposure and BPD in Psychodynamic Approaches

Introduced by psychoanalyst A. Stern in 1938, the term ‘borderline’ described a group of patients who ‘fit frankly neither into the psychotic nor into the psychoneurotic group.’ In the psychoanalytic theory of borderline genesis, emphasis was placed on the idea of an early trauma occurring at the anal stage and altering the later ability of the infant to access to more mature stages of the libido, based on Freud’s early theory of trauma (Freud, 1905). The nature of the trauma was later extended, theoretically, to repeated frustration, or repeated exposure to violence (Kernberg, 1967), and remained in the more recent psychoanalytic works (Masterson, 1981). In the psychoanalytic model, the ‘interpersonal relationships’ dimension of BDP is related to the developmental failure of a so called stable ‘object relationship.’ In terms of psychoanalytic treatment, it is proposed to offer during therapy transference interpretation to the patient (Høglend, 2014).

Later, Linehan developed the idea of the invalidating environment, according to which the emotional experience of the infant was never validated, supported, acknowledged by the caregivers (Linehan et al., 1994). This is in line with the strong association of BPD with insecure attachment in the context of attachment theory – 92–96% of patients with BPD are coded as insecure (Levy, 2005).

Altogether, these models based on clinical investigations and experience insist on: (i) the occurrence of adversity early in childhood, (ii) the lacking of support for the infant to process these inappropriate stimuli, and (iii) later be unable to develop emotional regulation and mentalization skills.

### Epidemiological Data

Empirically, clinical observations have emphasized the existence of traumatic episodes in the past histories of subjects exhibiting the symptoms of BPD. Numerous epidemiological studies have found a significant statistical association between BPD and past traumatic experiences (Herman et al., 1989; Ogata et al., 1990; Zanarini et al., 1997; Bierer et al., 2003). This association is now considered part of the BPD picture. From the largest study (*N* = 358 individuals with BPD), 91% of BPD patients reported having been abused and 92% reported having been neglected before the age of 18 (Zanarini et al., 1997). Overall, traumatic events were found in 70.7% of patients with BPD, among which were emotional neglect (43.7%), witnessing violence (43.0%), physical abuse (36.4%), sexual abuse (25.8%), and physical neglect (9.3%). Compared to patients with other personality disorders, those with BPD significantly reported having been emotionally or physically abused by a caretaker or sexually abused by a non-caretaker. When all the significant risk factors were considered together (multivariate analysis), four factors remained significant predictors of BDP: female gender, sexual abuse by a male non-caretaker, emotional denial by a male caretaker, and inconsistent treatment by a female caretaker (Zanarini et al., 1997). In a later study, Zanarini reported that 84% of people with BPD retrospectively described experience of biparental neglect and emotional abuse before the age of 18, with emotional denial of their experiences by their caregivers as a predictor of BPD (Zanarini, 2000).

From a developmental perspective, the timing of trauma has been shown to play a modulating role in brain changes, behavior and cognition (Lupien et al., 2009). Early occurrence of the traumatic experiences seems to increase the risk of developing BPD (Ogata et al., 1990; Zanarini et al., 1997; Johnson et al., 1999). More precisely, Johnson et al. (1999) found a significantly higher prevalence of abuse or neglect occurring before the age of 10 in individuals with BPD [OR = 7.75 (1.78–33.48)].

### Traumatic Past and Nosography: Are BPD and PTSD the Same Disorder?

Samples from many studies show high rates of comorbidity between BPD and PTSD: 24–68% of PTSD patients also have BPD (Heffernan and Cloitre, 2000; Zlotnick et al., 2002; Pagura et al., 2010), and 25–39% of BPD subjects also meet the criteria for PTSD (Zanarini et al., 1997; Golier et al., 2003; Pagura et al., 2010). Additionally, several studies found that “BPD + PTSD” comorbidity was more frequently associated to a history of trauma when compared to BPD alone (Zlotnick et al., 2002) or to PTSD alone (Connor et al., 2002). Among individuals with both disorders compared to individuals with PTSD only, trauma may have more severe features: history of sexual abuse occurs earlier and there is a higher frequency of physical and verbal abuse by the mother (Heffernan and Cloitre, 2000; Clarke et al., 2008). Additionally, co-occurrence of BPD and PTSD correlates with an impaired quality of life, more Axis I comorbid disorders (as described in the DSM-IV), and more suicide attempts (Bolton et al., 2006; Pagura et al., 2010).

Common clinical features can be found such as episodes of dissociative states, mood lability, behavioral problems such as irritability and aggressivity, and their relational consequences.

The conceptual frame of Complex PTSD (c-PTSD) was developed by J. Herman and encompasses “all persistent psychological reactions secondary to usually severe and prolonged or repeated stressors” (Herman, 1992; Cloitre et al., 2009). Latent class analysis allows a clear distinction between BPD and c-PTSD (Cloitre et al., 2014): (i) Early traumatic experiences are not necessary to establish a BPD diagnosis; (ii) fear of abandonment or distorted self-image are specific to BPD; (iii) relationship problems exist in both clinical situations but are supposed to be underpinned by a chaotic attachment in individuals with BPD and by social avoidance in c-PTSD; and (iv) expression of emotional instability differs; specifically, heightened reactivity, mismatched answers, irritability and anger are found in both cases, but self-injurious behavior, including suicide attempts, is specific to BPD.

Finally, BPD and c-PTSD are both associated with a common traumatic past; however, their symptomatic expressions show important differences that also suggest patho-psychological differences (Frias et al., 2016). In BPD individuals early trauma is associated with fear of abandonment, whereas patients with PTSD experience traumatic resurgences. Recently, a developmental hypothesis was formulated to explain both the vicinity and differences between the two disorders: BPD would most likely develop when the trauma occurs early in life, whereas PTSD (or c-PTSD) would develop in a more mature brain (Amad et al., 2016).

### Is Trauma a Causal Factor in BPD Symptomatology?

Despite the statistical association, causality between childhood trauma and the emergence of BPD is not obvious. It is still a matter of debate (Paris, 1997). Ball and Links reviewed several articles published between 1995 and 2007 discussing this causal link and analyzed them using the Hill criteria which allow linking a statistical association to a causal relation. The criteria include strength of association, temporality, dose-response effect, specificity, reproducibility, analogy, and epidemiological and biological plausibility. When studied within this methodological frame, childhood trauma may be considered a cause of later BPD when included in a multifactor aetiological model that accounts for the lack of specificity and robustness of the statistical association (Ball and Links, 2009). This causal link was also discussed in the light of its lack of specificity. Indeed, early traumatic events are a common risk factor for many disorders, both psychic and somatic (Johnson et al., 1999; Golier et al., 2003; Norman et al., 2012). Chronic exposure to stress is known to have deleterious effects on general functioning (e.g., immune alterations, atherosclerosis, obesity, bone loss, and neuronal atrophy) and cognitive functioning (e.g., learning ability and memory via the hippocampus, amygdala, and prefrontal functional changes) (Danese et al., 2007). Conversely, traumatic history is not always found in patients with BPD. Finally, BPD cannot be considered a necessarily or exclusively traumatic entity from an anamnestic point of view.

### Do Past Trauma and Current BPD Symptoms Share a Common Vulnerability?

Another hypothesis to explain the statistical association between BPD and trauma is the existence of a confounding factor, a common vulnerability (Brostedt and Pedersen, 2003; Pagano et al., 2004). Recent exposure to stressors was found to be specifically associated with BPD relative to other mental disorders – in particular, recent exposure to interpersonal conflicts or other negative life events (Pagano et al., 2004; Wingenfeld et al., 2011). This finding raises the issue of common mechanisms of occurrence.

According to the Big Five model, some personality traits – such as neuroticism – may promote both exposure and sensitivity to traumatic events (Breslau et al., 1995). Individuals with a strong neuroticism component tend to interpret surrounding stimuli as threatening, are prone to negative cognitive assessments when confronted with stressful events, thus experiencing a stressor as more stressful than it is for others (Jovev, 2006), and finally select ineffective adaptive strategies (Bolger and Schilling, 1991). Stress exposure is further increased because these subjects experiment with situations where interpersonal stressful events are more likely to occur. Eventually, personality disorders characterized by a high level of neuroticism and psycho-social dysfunction, especially in relationships, such as BDP (Jovev, 2006), are associated with frequent exposure to stressors and difficulty responding to them.

A recent study (Bornovalova et al., 2013) based on a cohort of twins examined whether childhood traumatic experiences (sexual, physical or emotional) may be considered causal. Internalized (e.g., depression and anxiety) or externalized (e.g., impulsivity) childhood disorders were also considered and introduced in the model as modulating developmental factors. Childhood abuse, BPD traits, and internalizing and externalizing symptoms were all correlated; however, the discordant twin analyses and biometric modeling showed little to no evidence of a causal effect of childhood abuse on the future emergence of BPD. This work rather tends to provide evidence that the statistical association between childhood abuse and BPD symptoms stems from a common genetic vulnerability that may also overlap with internalizing and externalizing disorders. However, the lack of measures regarding attachment style limits the conclusion of the study as many authors consider disorganized attachment style a key modulating factor in BPD (Baryshnikov et al., 2017).

### Current Candidates for the _“_Missing Link_”_

#### Early Disturbed Attachment: Oxytocin

Several studies have shown that oxytocin is a key hormonal messenger for early infant/caregiver interaction, impact of early stress, attachment patterns, future response to acute stress, and non-genetic transmission of behavioral traits (Feldman, 2012). OXT has anxiolytic properties; however, it neither generates indifference to a fear stimulus (in contrast to sedatives) nor prevents one from learning conditioned fear. These results have been believed to open the door to therapeutic perspectives on patients with PTSD (Koch et al., 2014).

In BDP patients, Bertsch et al. (2013b) found significantly reduced OXT serum levels compared to those in controls. These rates were inversely correlated with childhood trauma history. However, a model integrating oxytocin as a mediator between trauma and BPD genesis could not be validated (Bertsch et al., 2013b). Studying the polymorphism of the gene coding for the OXT receptor gene, Hammen et al. (2014) found a significant association between some allelic variants (rs53576) and BPD occurrence in a large sample of 20-year-olds under the influence of family environment (depressed or non-depressed mothers). In particular, allele A carriers experienced a massive influence from family functioning “for better or for worse,” while homozygous GG development was less influenced.

The same group studied the influence of OXT on social cognition distortions in BPD patients (Bertsch et al., 2013a). They found that the heightened sensitivity to social threats (measured by eye-tracking and fMRI protocols in response to faces expressing fear and amygdala activation in fMRI) was significantly attenuated after administration of intranasal OXT. A prospective randomized double blind study using intranasal oxytocin versus placebo at the waning of a social stress task (Trier Social Stress Test: TSST) found a decreased dysphoric stress-induced feeling in BPD patients compared to controls and a lower plasma cortisol rate when OXT was administered (Simeon et al., 2011).

#### Vulnerability: Genetic Profile of the HPA Axis

The impact of genetic factors on the development of BPD has been highlighted by family and twin studies (Leichsenring et al., 2011). A pioneering study in the field revealed that a polymorphism (Val158Met) of the gene coding for Catechol-*O*-MethylTransferase was significantly associated with past traumatic events and impulsive tendencies in individuals with BPD (Wagner et al., 2010). Few studies have tackled the issue of the genetic equipment of the HPA axis in BPD subjects. Martín-Blanco et al. (2015) worked on a large sample of BPD patients and controls (481 subjects with BPD and 442 controls), analyzing 47 polymorphisms in 10 HPA axis genes. To take into account the possible modulation of genetic associations by the presence of childhood trauma the sample was divided into three groups: BPD with trauma, BPD without trauma and controls. Significant associations were found for the BPD + trauma group for given polymorphisms of FKBP5 – a gene known to be involved in post-traumatic vulnerability, anxiety and depression. As stated by Amad et al. (2014), these approaches are promising to give more insight into the comprehension of the pathophysiology of BPD (and PTSD), and may be further explored with imaging genetics studies, centered on connectivity analyses, or further epigenetic studies.

Taken together, these data show that BPD symptoms often occur in the context of a traumatic personal history and that they share clinical features with PTSD. Nevertheless, subtle symptoms seem to support a distinction between the disorders. Furthermore, after decades of research, the aetiological value of early trauma has never been quantitatively established. Newer studies taking a high level of complexity into account and the notion of shared vulnerability may elucidate the relationship between a traumatic past and current BPD.

## Stress Reactivity in BDP

In BPD subjects, stress may be part of the past but is also an experience of the present. Under stressful situations, clinical observations indicate that reactions may trigger extreme behavioral responses in BPD subjects. In this section, we review the experimental evidence characterizing BPD subjects’ responses to stress, distinguishing subjective experience, behavioral observations, and neuro-vegetative and hormonal measures.

### Perceptions of Stressful Stimuli and Subjective Experience

To date, few studies have addressed the issue of subjective experience in the context of stressful events in BPD subjects. *Pain* has been widely used as a paradigmatic stressful stimulus. Using cold application to trigger a nociceptive perception, Russ et al. (1992) found a higher sensitivity threshold to pain in patients with BPD compared to controls. This decreased sensitivity to pain was shown to be associated, in BPD individuals, with anxious, depressive, dissociative, and impulsive symptoms. Later, using laser-evoked potentials, Schmahl et al. (2004) confirmed that pain detection thresholds were increased in BPD patients compared to controls. This observation was then generalized to a population of female BPD adolescents, showing that disturbed pain processing is not only a consequence of chronic BPD but is already present in the early stages of BPD (Ludäscher et al., 2015). Nevertheless, while the threshold for the stimulation to be considered “painful” was higher in BPD subjects, the perception of the intensity of the stimulation was unchanged. Thus, the perception in itself does not seem to be altered but rather the subjective experience of pain, namely what triggers the shift from an uncomfortable sensation to a painful experience, recalling here that pain is a complex phenomenon associating a perceptive and emotional processing of the stimuli (Bonnot et al., 2009). The inner (self-reported) state of calmness or distress also participates in the pain threshold modulation in experimental settings. Even during self-reported calmness, patients with BPD showed a significantly reduced perception of pain compared to healthy controls (HC). During distress, pain perception in BPD patients was significantly further reduced compared with self-reported calmness (Bohus et al., 2000). Additionally, self-inflicted pain has been recently shown to have a direct soothing effect on stress manifestations in BPD subjects, an aspect that is discussed below (Naoum et al., 2016; Willis et al., 2016). In relation to our topic, it remains of course a matter of debate whether pain can be considered as an extreme version of stress and studied as such, or whether the specific involvement of the pain matrix system makes such parallels irrelevant. However, in the context of BDP, we believe that it may be relevant to the increased use of cutting in these patients.

In recent works, exposure to psychosocial stress was also used to study more specifically the issue of stress in BPD. From a phenomenological point of view, the TSST was shown to trigger enhanced negative emotions and negative cognitions in BPD subjects compared to HC (Deckers et al., 2015), whereas their physiological responses were attenuated. But when compared to Cluster C personality disorders (e.g., avoidant, dependent or obsessive-compulsive, as described in the former DSM4-R) no difference was observed in subjective experience with BPD subjects when the TSST was performed (Aleknaviciute et al., 2016).

### Behavioral Responses to Acute Stress

*Impulsivity* is a core feature of BPD, and in contrast to other psychiatric conditions that are also characterized by impulsivity (such as Attention Deficit Hyperactivity Disorder – ADHD), impulse control deficits in BPD occur specifically under stressful conditions. This clinical observation was investigated experimentally in BPD, ADHD and controls (Krause-Utz et al., 2016) showing that (i) both patient groups reported higher impulsivity than controls, (ii) ADHD reported higher trait impulsivity than BPD, (iii) action-withholding deficits were significantly increased under stress compared to baseline in BPD but not in ADHD and controls, and (iv) under stress only, BPD performed significantly worse than controls in action-withholding tasks, whereas ADHD always showed significant deficits (under stress or non-stress conditions). This experiment was conducted using a multicomponent stress task, and the results reveal the relative specificity of impulsivity states in BPD and in particular their causal and close temporal relationship to stressful situations.

A second feature is *non-suicidal self-injurious (NSSI) behavior.* Approximately 60–90% of patients with BPD display NSSI behavior, with cutting being the most frequently applied method. When interviewed in clinical settings, patients spontaneously relate NSSI to high levels of stress or anxiety and emphasize the tension-relieving effect of such behaviors (Corcos et al., 2013). Two recent studies by the same group (Naoum et al., 2016; Willis et al., 2016) investigated the respective effects of the eligible components of NSSI on stress-related arousal via pain, tissue damage and sighting of blood in both BPD and controls. This research shows that among BPD patients, the nociceptive input specifically leads to stress reduction. In contrast, the impact of tissue damage on stress reduction was relatively small (Willis et al., 2016), as was the impact of seeing blood (Naoum et al., 2016). Additionally, the results suggest that painful stimuli lead to greater stress reduction in BPD patients compared to Controls. Taken together, these results again show the complex and specific relationship between pain and stress in BPD: more than a stressor, acute pain is likely a stress reliever in this population, and NSSI behaviors are simultaneously stress-induced and stress-releasing.

### Neuro-Vegetative Reactivity

The Polyvagal Theory Model (Porges, 2009) provides crucial elements about the neurobiological substrate of adaptive social behavior. Through species evolution, three stages of development of the neuro-vegetative response to stress are supposed to have taken place, underpinning physiological and behavioral responses to acute stress: (i) development of the parasympathetic system, represented by the unmyelinated vagus nerve. In a stressful situation, its action is responsible for decreased metabolic activity and inhibited behavioral responses (freezing). (ii) The emergence of the sympathetic nervous system, underpinning the possibility of an activated behavioral response (“fight or flight”) in stressful situations. Finally, (iii) myelination (specific to mammals) increases the speed of regulation of neuro-vegetative output and allows faster and more subtle engagement or disengagement responses. The *vagal tone* represents the global functioning of the neuro-vegetative system, based on the parasympathetic/sympathetic balance. An increase in vagal tone (parasympathetic > sympathetic) both slows the heart and makes the heart rate more variable (i.e., there are more beat-to-beat changes between heartbeats), showing better subtle adaptive abilities. In experimental settings, vagal tone cannot be directly measured but is indirectly observed by measuring periodic changes in the heart rate during a resting state of cardiovascular activity, a process called Heart Rate Variability (HRV). Respiratory Sinus Arrhythmia (RSA) stands for the variations in heart rate associated with inhalation and exhalation times.

In response to stress, it has been hypothesized that BPD patients may show a neuro-vegetative imbalance, with an increased sympathetic response and a decreased parasympathetic response. This hypothesis would account for the disrupting behaviors that are more frequent in BPD (arousal of the “fight or flight” response). Recent work using indicators of neuro-vegetative functioning confirmed this hypothesis: in stressful conditions, BPD subjects show a low vagal tone (Weinberg et al., 2009). In a recent meta-analysis based on 5 studies (95 BPD subjects and 105 controls), Koenig et al. (2016) showed that decreased vagal tone is significantly associated with impulsivity and emotional liability, two clinical dimensions that are key but not specific to BPD diagnosis. Another recent study by Kuo et al. (2016) using markers such as heart rate and RSA showed a significant difference in vagal tone at baseline in BPD patients compared to controls.

Comorbid symptoms associated with BPD may modulate the neuro-vegetative response to an elicited stress. When BPD subjects are distinguished according to the level of Peritraumatic Dissociation (PD) based on the Peritraumatic Dissociative Experiences Scale, it appears that BPD with high PD (i) display the highest degrees of trauma exposure (ii) the most severe clinical symptoms, and (iii) a significant decrease in heart rate during stress elicitation. In contrast, an increase in heart rate was observed in the two other groups during stress elicitation (BDP with low PD and controls) (Bichescu-Burian et al., 2016). Subjects with PTSD were also found to have a lower vagal tone (measured by HRV) at baseline than BPD; however, the comorbidity was not studied (Meyer et al., 2016). Other markers of neuro-vegetative response to stress (e.g., galvanic skin response) have not been investigated in BPD.

### Acute Hormonal and Enzymatic Reactivity

Stress response patterns may also be identified through biomarkers. *Salivary alpha amylase* appears to be a non-invasive enzymatic biomarker of sympathetic nervous system activity that is well correlated with cardiovascular parameters (Nater and Rohleder, 2009) that increases after a specific activation of the sympathetic system but may also be a marker of parasympathetic activity. The other stress response system is the activation of the HPA axis (hypothalamic-pituitary-adrenal), which results in ACTH and glucocorticoid secretion. Thus, s*alivary cortisol* is a non-invasive hormonal biomarker of the activity of the HPA axis and faithfully reflects free cortisol plasma levels.

In BPD patients, *basal cortisol* is higher than in controls, whether using repeated diurnal measurements in saliva (Lieb et al., 2004) or three consecutive nocturnal urine samples (Wingenfeld et al., 2007a). After a psycho-social stress, alpha amylase and cortisol responses are attenuated despite a greater subjective stress experience reported by BPD subjects (Nater et al., 2010; Scott et al., 2013). Several comorbid symptoms were identified as modulating the cortisol levels in individuals with BPD (Zimmerman and Choi-Kain, 2009). Depressive comorbidity is associated with higher basal cortisol levels, whereas a greater severity of PTSD symptoms is associated with lower basal cortisol rates (Wingenfeld et al., 2007b). In response to a stressful situation, BPD patients with severe PTSD symptoms showed a decreased cortisol response compared to that of BPD patients with less severe PTSD symptoms (Dixon-Gordon et al., 2013). Also, the negative feedback of the cortisol loop (evaluated by the suppression test with dexamethasone) is less intense in BPD compared to controls (Carvalho Fernando et al., 2012). When studied more precisely, this feedback is stronger when associated with a childhood trauma history and dissociative symptoms (Carvalho Fernando et al., 2012). Conversely, it is weaker when associated with depressive symptoms (Wingenfeld et al., 2007b).

**FIGURE 2:**
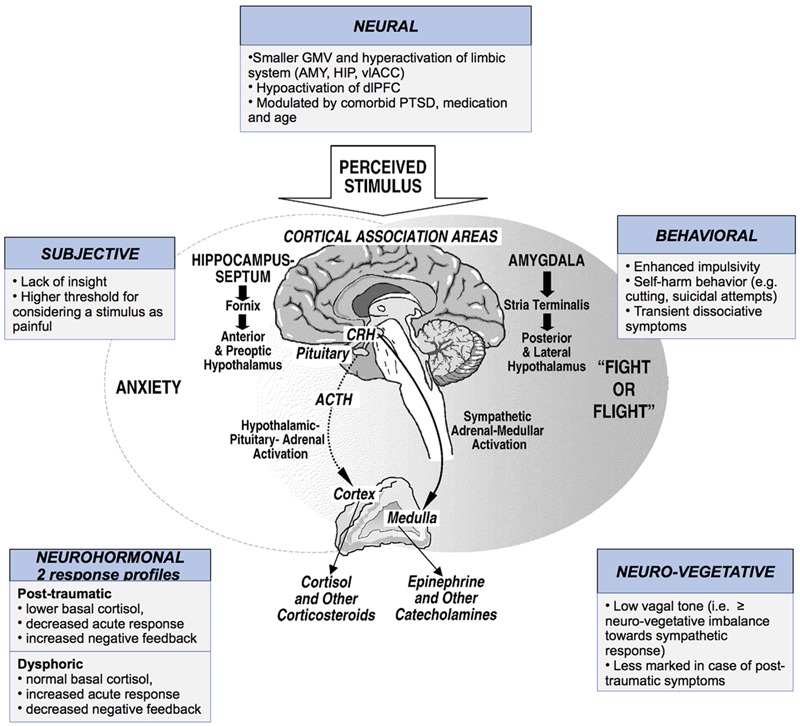
Stress response in borderline personality disorder (BPD) subjects according to subjective experience, behavior, neural, neuro-vegetative and neuro-hormonal changes. GMV, gray matter volume; AMY, amygdala; HIP, hippocampus; vlACC, ventrolateral anterior cingulate cortex; dlPFC, dorsolateral prefrontal cortex; PT, post-traumatic; CRH, cortisol-releasing hormone; ACTH, adrenocorticotrophic hormone.

In summary, based on the literature available, we may distinguish two groups of BPD patients by their pattern of biological stress reactivity: (i) The first is characterized by a severe traumatic experience and PTSD symptoms and shows reduced (or normal) basal cortisol level, reduced (or normal) cortisol response to stress, and increased negative feedback; (ii) the second includes patients with mood symptoms who show a high basal cortisol level, a high cortisol response to stress, and reduced negative feedback (Wingenfeld et al., 2010). These findings were summarized in **Figure 2**.

## Structural and Functional Imaging of Perception, Reactivity and Stress Regulation in BPD Patients

To our knowledge, no study has specifically investigated the neuroanatomical correlates of acute stress processing in BPD patients. However, neuroimaging studies on BPD focused on emotion perception and regulation known to be altered in this disorder. Most task-based fMRI studies used negative emotion stimuli. This type of paradigm can be interpreted as a ‘stressor’ and offers an opportunity to better understand the neurobiological underpinnings of stress reactivity in BPD.

A short insight of structural imaging studies are necessary to recall before addressing functional imaging. These studies focused on fronto-limbic structures known to be involved in stress perception, emotion processing and regulation (Pruessner et al., 2008; Wingenfeld et al., 2010; van Zutphen et al., 2015; Schulze et al., 2016). Reduced gray matter volume (GMV) of the hippocampus has been reported in BPD (in the right and, to a lesser extent, the left hippocampus) (Bøen et al., 2014; Krause-Utz et al., 2014; Schulze et al., 2016). Similar findings have been evidenced in PTSD and in healthy adults with traumatic history, and reduced hippocampal GMV may be interpreted as the result of trauma rather than a BPD specificity (Woon et al., 2010; Rodrigues et al., 2011; Ruocco et al., 2012; Krause-Utz et al., 2014). Nevertheless, two different studies found no correlation between hippocampal volume loss and Childhood Trauma Questionnaire (CTQ) scores (Kuhlmann et al., 2013; Bøen et al., 2014).

Reduced volume of the amygdala may be more specific to BPD. It has not been described in PTSD (Wingenfeld et al., 2010). Furthermore, a voxel-based study (Niedtfeld et al., 2013) found that BPD symptom severity predicted volume loss in the amygdala regardless of PTSD comorbidity. Chanen et al. (2008), comparing BPD adolescents to HC, found no difference between the volumes of the amygdala and hippocampus, suggesting that this volume loss may be a consequence of the disorder.

Finally, despite its crucial role in the HPA axis function, only one neuroimaging study focused on the hypothalamus. The results showed an increased GMV in the left hypothalamus in BPD compared to the GMV of HC which was positively correlated with CTQ scores but not with comorbid PTSD (Kuhlmann et al., 2013).

*Frontal regions involved in regulatory processes.* Wingenfeld et al. (2010) and Krause-Utz et al. (2014) showed a reduced GMV of the anterior cingulate cortex (ACC), whereas Kuhlmann et al. (2013) showed no difference in ACC volume between BPD and HC. These discrepancies might be due to the functional heterogeneity of the ACC, with its dorsal part known to be implicated in cognitive and regulation processes and its ventral part involved in emotion processing. More recently, Jin et al. (2016) showed significant bilateral GMV increases in the middle cingulate cortex (MCC) and the posterior cingulate cortex (PCC). A recent meta-analysis highlighted a relatively greater GMV in the right middle frontal gyrus (BA9), which is part of the dorso-lateral prefrontal cortex (dlPFC) and is involved in the inhibition of emotions and unwanted memories (Kluetsch et al., 2012), a function that may be “over-used” in BPD (Schulze et al., 2016). Furthermore, Krause-Utz et al. (2014) also reported reduced volume in the orbito-frontal cortex (OFC). Altogether and despite discrepancies, these structural results tend toward a decrease in the GMV of the structures involved in emotions and stress processing and an increase in the GMV of the regions involved in inhibition and regulation processing.

### Functional Activity and Connectivity of Stress Perception and Regulation in BPD

Most functional fMRI studies in the field used passive viewing of emotional faces or social scenes as emotional stimuli. Their most consistent finding was greater and prolonged activation of the right amygdala and hippocampus to stressful stimuli in medication-free BPD patients and decreased activity in the bilateral dlPFC and ACC (Minzenberg et al., 2007; Koenigsberg et al., 2009; Schulze et al., 2011, 2016; Hazlett et al., 2012; Bertsch et al., 2013b; Nicol et al., 2015; van Zutphen et al., 2015). A multimodal study also reported amygdala “hyperconnectivity” in BPD during both emotional challenge and resting state fMRI (Salvador et al., 2016).

More recently, Reitz et al. (2015) conducted a study using the Montreal Imaging Stress Task (MIST Task), which combines arithmetic with an algorithm causing disappointment that is known to induce social stress in HC (Dedovic et al., 2005). The task was associated with either an incision into the forearm (NSSI) or a sham treatment following stress induction. From a behavioral perspective and as previously described, stress was significantly reduced by NSSI and was significant in the BPD group compared to HC. Furthermore, the amygdala activity decreased more in BPD than in HC, and the connectivity of the amygdala with the superior frontal gyrus was increased in BPD after the incision compared with the sham treatment, whereas the control group showed reduced connectivity in response to the incision. Whereas the incision seems to alter emotion regulation in healthy controls, it can be interpreted in BPD as an attempt to cope with an enhanced stress sensitivity.

A few fMRI studies also focused on transient stress-related dissociation which occur in about 75–80% of BPD patients, with an intensity directly correlated to self-reported stress-levels (Stiglmayr et al., 2008). These studies found diminished amygdala reactivity and increased activity in left inferior frontal gyrus during emotion processing. Although the neurobiological underpinnings of state/trait dissociation remain unclear, it may be interpreted as a maladaptive coping strategy to face acute stress in BPD patients (Ludäscher et al., 2015; Schmahl and Baumgärtner, 2015; Krause-Utz et al., 2016). The two latter profiles of amygdala reactivity in BPD may be related to the two previous patterns of biological stress reactivity (see section “Stress Reactivity in BDP”): (i) an increase of amygdala response in the context of social stress that can be reversed by NSSI behavior; (ii) a decrease of amygdala response in a more severe state of stress response clinically associated with dissociative symptoms.

## Conclusion

**FIGURE 3:**
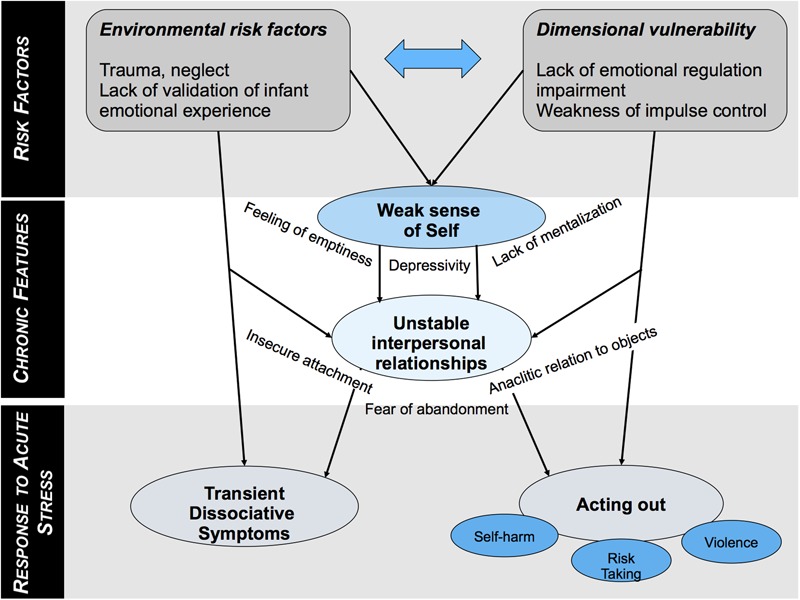
Putative relationships between risk factors and emergence of symptoms in BPD distinguishing chronic features and acute response to stress.

Stress and BPD have complex relationships. Epidemiological data partially confirm the early psychoanalytic theory, showing a strong statistical association between past trauma and BPD. BPD also shares clinical features with PTSD, although patients with BPD also present clear specificities, and trauma is not necessary to develop BPD. Direct causality between past traumatic events and outbursts of BPD symptomatology has never been proven formally, and recent work rather support models that associate individual genetic vulnerability and the experience of early adversity in development. The features of this stressful time are possibly various, may not consist in unique or discrete events, and consequently may not be easily detectable by questionnaires or labeled as traumatic. In contrast, these experiences are often related to inappropriate interactions with the close caregiving environment, which may cause disturbed attachment, dysfunction of the OXT axis, and underlie the specificity of later unstable relationships patterns of BPD subjects. We propose a synthetic figure to summarize the putative relationships between the risk factors and the emergence of symptoms of BPD (**Figure 3**).

Additionally, individuals with BDP display many dysfunctions that alter their response to stress at multiple levels. We sought to summarize in **Figure 3** the main findings of this review. First, BPD subjects show specific patterns of response to acute stress. They show an imbalance in neuro-vegetative response where sympathetic activation is privileged, which may underpin their impulsivity and behavioral arousal under such circumstances. The acute hormonal response is modulated by co-occurring symptoms that may help define groups within the BPD spectrum. In particular, patients with a “post-traumatic profile” tend to have a low cortisol baseline, a diminished cortisol reactivity to stress, and an enhanced negative retro-control loop. These differences find echoes in imaging studies. Second, pain, a common powerful stressor in the general population, may act as a stress reliever in BPD subjects; pain may act as a stressor and as a stress reliever, although the underlying mechanisms remain unknown.

This synthetic approach suggests distinguishing two types of acute stress response in BPD. The first profile is characterized by frequent NSSI behaviors; increased cortisol response and decreased vagal tone in response to stress; increased response of the amygdala to social stress. This profile may be related to impulsive subjects. The second profile is characterized by less self-harm behavior and a tendency to dissociative states; lower cortisol baseline and a stronger retroactive loop; an increased vagal tone in response to a stressful stimuli; a decreased response of the amygdala when dissociative states are attained. This profile may be related to more dysphoric or internalizing subjects.

In the collective work to write the new proposals for the DSM-5, the position of personality disorders has been a topic of intense debate (Robin and Rechtman, 2014). The former structure of the DSM4, divided into axis, was suppressed. Proposals were made to deeply change the diagnosis frame for personality disorders, and adopt preferentially a dimensional approach (Guelfi, 2014). Though Kruegers work on dimensional approach actually stands in the new version of the manual (DSM-5), the categorical nosography was maintained in the current classification since evidence for the benefits of a classification change were lacking. In line with this dimensional approach, we propose here that stress response profile may be considered as a dimension along which BPD may be studied.

Though BPD may be a useful diagnosis in terms of psychopathology, specific and *trans*-nosographic work on emotional regulation in people with BPD, depression or anxiety disorders have already been tested and proven effective. Knowing that this dimension is sensitive to focused therapy, a specific work on stress may be developed taking into account specificities related to the vegetative/hormonal/neural response. Such an approach based on objective measurements may help to refine the use of bio- or neurofeedback techniques for these patients where the work with the more personalized profiles is always beneficial. Thus, addressing the issue of stress in BPD through further multimodal and experimental settings may help to further explore whether response to stress can be considered as a relevant dimension to better understand this fascinating and complex condition, and to propose refined therapeutic options to the subjects.

## Author Contributions

NB and DC conceived the review. NB, CC, and AA analyzed, reviewed the papers, drafted the manuscript and designed the synthetic diagrams. DC revised the manuscript. All authors read and approved the final manuscript.

## Conflict of Interest Statement

The authors declare that the research was conducted in the absence of any commercial or financial relationships that could be construed as a potential conflict of interest. The reviewer PV and handling Editor declared their shared affiliation.
